# Efficacy and safety analysis of TACE + sunitinib vs. sunitinib in the treatment of unresectable advanced renal cell carcinoma: a retrospective study

**DOI:** 10.1186/s12885-023-10754-0

**Published:** 2023-03-24

**Authors:** Haohao Lu, Qing Ye, Chuansheng Zheng, Li Fan, Xiangwen Xia

**Affiliations:** 1 Department of Radiology, Union Hospital, Tongji Medical College, Huazhong University of Science and Technology, Jiefang Avenue #1277, Wuhan, 430022 China; 2 Hubei Province Key Laboratory of Molecular Imaging, Wuhan, 430022 China; 3 Department of Oncology, Union Hospital, Tongji Medical College, Huazhong University of Science and Technology, Jiefang Avenue #1277, Wuhan, 430022 China; 4 Huazhong University of Science and Technology Hospital, Luoyu Road #1037, Wuhan, 430071 China

**Keywords:** Renal cell carcinoma, Molecular targeted therapy, Transcatheter arterial chemoembolization, TACE, Renal artery embolization, Sunitinib, Transarterial embolization

## Abstract

**Background:**

Since renal cell carcinoma(RCC) is insensitive to conventional chemoradiotherapy, molecularly targeted drugs are commonly used treatments for unresectable advanced RCC. The aim of this study was to explore the efficacy and safety of TACE + sunitinib vs. sunitinib in the treatment of unresectable advanced RCC.

**Methods:**

This study included 98 patients with unresectable advanced RCC who were treated in Union Hospital from January 2015 to December 2018, and they met the criteria. They were divided into two groups: TACE + Sunitinib group (N = 47) and Sunitinib group (N = 51). We conducted a retrospective study to analyze the efficacy and safety of the two groups of patients.

**Results:**

(1)TACE + Sunitinib group: 4 patients (8.5%) achieved CR, 27 patients (57.5%) achieved PR, 9 patients (19.1%) achieved SD, and 7 patients (14.9%) achieved PD. Sunitinib group, 0 patients (0%) achieved CR, 20 patients (39.2%) achieved PR, 14 patients (27.5%) achieved SD, and 17 patients (33.3%) achieved PD. (P = 0.017) (2)ORR: TACE + sunitinib group, 66.0%; sunitinib group, 39.2%. (P = 0.009) (3)DCR: TACE + sunitinib group, 85.1%; sunitinib group, 66.7%. (P = 0.038) (4) In the TACE + sunitinib group, mPFS was 15.6 months, mOS was 35.0 months; in the sunitinib group, the mPFS was 10.9 months, mOS was 25.7 months. (P < 0.001) (5) The incidence of abdominal pain, fever, and vomiting was higher in the TACE + sunitinib group than in the sunitinib group (abdominal pain: 55.3% vs. 13.7%; fever: 61.7% vs. 7.8%; vomiting: 40.4% vs. 19.6%; P < 0.05). The technical success rate of TACE in TACE + Sunitinib group is 100%.

**Conclusions:**

The TACE + sunitinib group had higher ORR and DCR, longer OS and PFS than the sunitinib alone group. TACE combined with sunitinib can play a complementary role and is a safe and effective treatment for advanced RCC.

Renal malignant tumor is one of the common tumors of the urinary system, and its incidence accounts for 3% of adult malignant tumors. The incidence of renal cell carcinoma(RCC) is lower than that of prostate and bladder cancer, accounting for the third most common urinary system tumor [1, 2]. Renal cell carcinoma(RCC) is a malignant tumor arising from the tubular epithelium, accounting for 80–90% of renal malignancies [3]. The causes of RCC are not fully clarified and may be related to genetics, smoking, obesity, hormone levels, hypertension and antihypertensive drugs, diet and occupational environment [4]. Clear cell renal cell carcinoma (ccRCC) is the most common pathological type (80%) [5], followed by papillary renal cell carcinoma (10% ~ 15%) [6] and chromophobe cell carcinoma (5%) [7]. For early RCC without metastasis, surgical resection of the primary tumor is still the most effective treatment [8]. However, approximately 30% of patients with RCC already present with distant metastasis at presentation. Meanwhile, 20–40% of patients with localized RCC will develop distant metastasis after surgery [9]. The biological behavior of RCC is also complex and diverse, and invasion of the venous system is one of its unique biological behaviors [10], and the incidence of venous tumor thrombus accounts for 5–15% of RCC, with renal venous tumor thrombus being the most common, accounting for 60–78% of venous tumor thrombus [11]. Renal vein tumor thrombus can further progress to the vein or even the right atrium. Because renal cell carcinoma is insensitive to traditional chemoradiotherapy, molecular targeted drugs and immunotherapy are commonly used treatments for unresectable advanced RCC [12, 13]. Commonly used molecular targeted drugs for the treatment of RCC are sunitinib, sorafenib, and pazopanib, which are multi-targeted tyrosine kinase inhibitors. However, targeted drugs often experience drug resistance and disease progression after a period of use, affecting the survival of patients. In recent years, immune checkpoint inhibitors have also been applied in the treatment of advanced renal cell carcinoma, and a number of randomized controlled studies have been conducted, obtaining positive findings. In the phase 3 CheckMate 214 trial [14], nivolumab plus ipilimumab led to improved efficacy outcomes versus sunitinib in both intermediate-risk/poor-risk patients that were maintained through 42 months’ minimum follow-up, with manageable safety. Nirmish Singla et al. [15] reported that cytoreductive nephrectomy combined with immunotherapy had a longer OS than immunotherapy alone for metastatic renal cell carcinoma (mOS NR vs. 11.6 months; hazard ratio 0.23, P < 0.001). Transarterial chemoembolization (TACE) was first proposed by Yamada in 1978 and is a commonly used treatment for advanced hepatocellular carcinoma [16]. The main principle of TACE is the transcatheter injection of chemotherapeutic agents and embolic agents into the tumor tissue. On the one hand, the cytotoxicity of chemotherapeutic drugs can induce the apoptosis of tumor cells and inhibit the proliferation of tumor cells; on the other hand, after embolization of tumor vessels, tumor tissue is ischemic and hypoxic and necrotic. In recent years, with the continuous update of interventional devices, embolization materials and operation techniques, TACE is also used for the treatment of multiple solid tumors throughout the body. There have been previous reports on preoperative combined TAE for RCC, and TAE has clinical significance for reducing the occurrence of complications such as intraoperative bleeding during surgery [17]. As one of the common solid tumors, some scholars have also tried TACE for RCC [18]. The aim of this study was to explore the efficacy and safety of TACE combined with sunitinib vs. sunitinib in the treatment of unresectable advanced renal cell carcinoma.

## Materials and methods

### General information

**Fig. 1:**
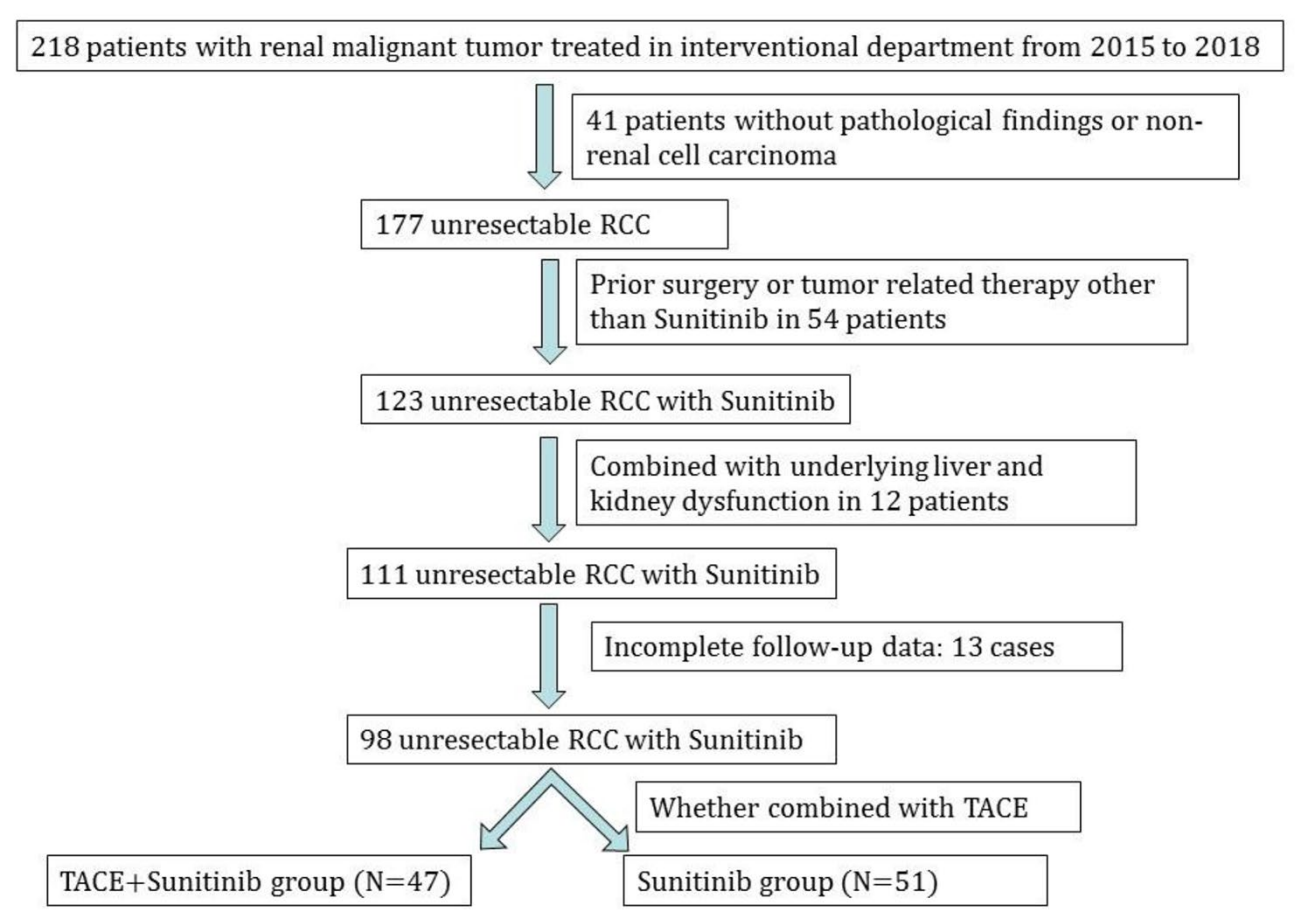
Flow Chart of Patient Enrollment

This study retrospectively analyzed the clinical data of 98 patients with unresectable advanced renal cell carcinoma treated in Union Hospital from January 2015 to December 2018. Inclusion criteria (1) RCC confirmed by pathological examination; (2) aged 18–75 years old; (3) with venous invasion or distant metastasis, surgical resection is not possible; (4) liver function classification Child-Pugh A-B, physical score (ECOG) 0–2 points; (5) normal renal function; (6) white blood cells ≧ 3.5G/L, platelets ≧ 100G/L, hemoglobin ≧ 100 g/L; (7) normal heart function, coagulation function; (8) complete clinical follow-up data. Exclusion criteria : (1) previously received other tumor-related treatment; (2) allergic to iodine contrast medium and sunitinib. All patients were treated with Sunitinib and divided into two groups according to whether they were combined with TACE or not: TACE + Sunitinib group (N = 47) and Sunitinib group (N = 51). A flow chart of patient enrollment is shown in Fig. 1. Baseline data were collected, including gender, age, pathological type, venous invasion, distant metastasis, IMDC risk classification, preoperative Child-Pugh classification of liver function, ECOG score, total bilirubin, albumin, Blood urea nitrogen(BUN), creatinine(Cr), glomerular filtration rate(GFR), white blood cells(WBC), red blood cells(RBC), and platelets(PLT).

### Method

#### TACE process

The patient was placed in the supine position, the inguinal region was disinfected, and a sterile drape was draped. Local anesthesia was performed at the puncture site using 2% lidocaine, the femoral artery was punctured using the Seldinger technique, and a 5 F catheter sheath was placed. A 5 F Yashino catheter was inserted into the renal arteries (accessory renal arteries, lumbar arteries if necessary) for arteriography to identify the feeding artery to the tumor. A 2.7 F microcatheter was then used to superselectively cannulate into the RCC feeding artery, inject the emulsion formed by mixing lipiodol + doxorubicin, and finally inject gelatin sponge particles. The dosage of doxorubicin was 30-50 mg, and the end point of embolization was stagnation of blood flow in tumor feeding arteries. If intraoperative angiography revealed combined arteriovenous fistula, superselective catheterization was performed to the fistula, PVA particle embolization was given to occlude the fistula, and subsequent chemoembolization was performed. At the end of the treatment, the catheter was removed and the puncture site was pressurized and dressed. Patients receive enhanced CT or MRI every 3–6 months, and decide whether TACE should be performed again according to re-examination conditions.

#### Treatment with Sunitinib

Usage: 50 mg, qd, oral, 4/2 regimen (4 weeks of medication, 2 weeks of drug withdrawal). If the patient has grade 3–4 adverse events, the sunitinib dose will be halved.

### Outcome measures

Primary study endpoints: Overall survival (OS) and progression-free survival (PFS) in the two groups;

Secondary study endpoints: The patients were evaluated after 3 months of treatment. (1) two groups of patients were evaluated for tumor response after treatment, and the imaging data (enhanced CT or MRI) of patients were evaluated using the mRECIST criteria [19], including complete response (CR), partial response (PR), stable disease (SD), progressive disease (PD); (2) two groups of patients with objective response rate (ORR), disease control rate (DCR); (3) changes of liver function, renal function, ECOG and blood routine before and after treatment in the two groups; (4) occurrence of treatment-related adverse events(AEs) in the two groups;

CR: all target lesions disappeared, no new lesions appeared, and maintained for at least 4 weeks. PR: the sum of the maximum diameters of the target lesions decreased by ≧ 30%, and maintained for at least 4 weeks. SD: the sum of the maximum diameters of the target lesions does not shrink to PR, or increase to PD. PD: the sum of the maximum diameters of the target lesions increases by ≧ 20%, or new lesions appear. ORR is the proportion of patients with CR and PR. DCR is the proportion of patients with PR + CR + SD.

### Statistical methods

Statistical analysis was performed using SPSS software(Version24.0, IBM, Armonk, NewYork). Number of cases (percentage) was used for enumeration data, and chi-square test was used for differences, including Pearson Chi-Square and Fisher’s Exact Test. Measurement data were expressed as mean ± standard deviation, and t-test was used for differences. OS and PFS were shown by Kaplan-Meier curves, and the Log-Rank test was used to compare OS and PFS between the two groups. P < 0.05 was considered to indicate a statistically significant difference.

## Results

### Baseline data of patients in the two groups(Table 1)

**Table 1:** Comparison of baseline data before treatment between the two groups

			Group		Chi-Square Tests(p-value)		t-test(p-value)
			TACE + Sunitinib group(N = 47)	Sunitinib group(N = 51)	Pearson Chi-Square	Fisher’s Exact Test	
Gender	Female	Count(%)	11(23.4%)	10(19.6%)		0.806	
	Male	Count(%)	36(76.6%)	41(80.4%)			
Pathological type	Clear cell carcinoma	Count(%)	39(83.0%)	40(78.4%)	0.802		
	Papillary renal cell carcinoma	Count(%)	5(10.6%)	6(11.8%)			
	Chromophobe cell carcinoma	Count(%)	3(6.4%)	5(9.8%)			
Venous tumor thrombus	None	Count(%)	16(34.0%)	21(41.2%)		0.534	
	Yes	Count(%)	31(66.0%)	30(58.8%)			
Distant metastasis	None	Count(%)	10(21.3%)	15(29.4%)		0.487	
	Yes	Count(%)	37(78.7%)	36(70.6%)			
Pre-treatment ECOG	0	Count(%)	17(36.2%)	18(35.3%)	0.490		
	1	Count(%)	24(51.1%)	22(43.1%)			
	2	Count(%)	6(12.8%)	11(21.6%)			
Pre-treatment liver function	Child A	Count(%)	38(80.9%)	40(78.4%)		0.807	
	Child B	Count(%)	9(19.1%)	11(21.6%)			
IMDC risk classification	Low Risk	Count(%)	26(55.3%)	24(47.1%)	0.664		
	Intermediate risk	Count(%)	18(38.3%)	22(43.1%)			
	High risk	Count(%)	3(6.4%)	5(9.8%)			
Age(Years)	Mean ± SD		58.5 ± 9.1	55.8 ± 12.9			0.245
Pre-treatment bilirubin(µmol/L)	Mean ± SD		12.3 ± 2.6	11.4 ± 3.0			0.124
Pretreatment Albumin(g/L)	Mean ± SD		37.62 ± 2.42	38.42 ± 3.37			0.182
Pretreatment BUN(mmol/L)	Mean ± SD		6.34 ± 1.43	6.07 ± 1.41			0.352
Pretreatment Cr(µmol/L)	Mean ± SD		87.4 ± 21.1	89.4 ± 17.7			0.606
Pretreatment GFR(ml/min)	Mean ± SD		107.06 ± 8.29	104.58 ± 8.81			0.155
Pretreatment WBC(G/L)	Mean ± SD		4.43 ± 0.72	4.63 ± 0.62			0.133
Pretreatment RBC(T/L)	Mean ± SD		4.39 ± 0.44	4.53 ± 0.58			0.179
Pretreatment PLT(G/L)	Mean ± SD		128.81 ± 19.93	124.67 ± 20.31			0.312

In TACE + Sunitinib group, the duration of treatment was 18–37 months (median:28 months), and the duration of follow-up was 16–49 months (median:37 months). In Sunitinib group, the duration of treatment was 10–35 months (median:21 months), and the duration of follow-up was 10–41 months (median:29 months). There were no statistical differences between the two groups in gender, age, pathological type, venous tumor thrombus, distant metastasis, pretreatment ECOG score, pretreatment liver function grade, IMDC risk classification, pretreatment bilirubin, pretreatment albumin, pretreatment BUN, pretreatment Cr, pretreatment GFR, pretreatment WBC, pretreatment RBC, and pretreatment PLT.(Table 1, P > 0.05).

### Changes of liver function and blood routine before and after treatment in the two groups

(1) Comparison of liver function and blood routine after treatment between the two groups(Table 2).

**Table 2:** Comparison of liver function, renal function, blood routine and performance status after treatment between the two groups

			Group		Chi-Square Tests(p-value)		t-test(p-value)
			TACE + Sunitinib group(N = 47)	Sunitinib group(N = 51)	Pearson Chi-Square	Fisher’s Exact Test	
Post-Treatment ECOG	0	Count(%)	11(23.4%)	11(21.6%)	0.570		
	1	Count(%)	24(51.1%)	22(43.1%)			
	2	Count(%)	12(25.5%)	18(35.3%)			
Post-treatment liver function	Child A	Count(%)	32(68.1%)	33(64.7%)		0.831	
	Child B	Count(%)	15(31.9%)	18(35.3%)			
Post-treatment bilirubin(µmol/L)	Mean ± SD		16.1 ± 4.8	15.0 ± 5.3			0.306
Post-treatment Albumin(g/L)	Mean ± SD		35.76 ± 5.41	33.37 ± 3.05			0.008
Post-treatment BUN(mmol/L)	Mean ± SD		7.71 ± 0.89	7.54 ± 0.98			0.347
Post-Treatment Cr(µmol/L)	Mean ± SD		110.3 ± 20.8	105.0 ± 25.6			0.269
Post-Treatment GFR(ml/min)	Mean ± SD		95.78 ± 12.97	99.34 ± 12.46			0.168
Post-Treatment WBC(G/L)	Mean ± SD		6.92 ± 0.72	3.52 ± 0.73			< 0.001
Post-Treatment RBC(T/L)	Mean ± SD		3.64 ± 0.66	3.37 ± 0.74			0.062
Post-Treatment PLT(G/L)	Mean ± SD		95.40 ± 18.03	87.90 ± 23.32			0.080

Compared with the TACE + sunitinib group and sunitinib group, albumin after treatment was 35.76 ± 5.41 vs. 33.37 ± 3.05 g/L (P = 0.008), and WBC after treatment was 6.92 ± 0.72 vs. 3.52 ± 0.73 G/L (p < 0.001), with statistical difference.

(2) The changes of liver function and blood routine before and after treatment were compared in each group;(Table 3).

**Table 3:** Comparison of liver function, renal function and blood routine before and after treatment in each group group

		Before treatment	Post Treatment	t-test(p-value)
TACE + Sunitinib group	Bilirubin(µmol/L)	12.3 ± 2.6	16.1 ± 4.8	< 0.001
	Albumin(g/L)	37.62 ± 2.42	35.76 ± 5.41	0.091
	BUN(mmol/L)	6.34 ± 1.43	7.71 ± 0.89	< 0.001
	Cr(µmol/L)	87.4 ± 21.1	110.3 ± 20.8	< 0.001
	GFR(ml/min)	107.06 ± 8.29	95.78 ± 12.97	< 0.001
	WBC(G/L)	4.43 ± 0.72	6.92 ± 0.72	< 0.001
	RBC(T/L)	4.39 ± 0.44	3.64 ± 0.66	< 0.001
	PLT(G/L)	128.81 ± 19.93	95.40 ± 18.03	< 0.001
Sunitinib group	Bilirubin(µmol/L)	11.4 ± 3.0	15.0 ± 5.3	< 0.001
	Albumin(g/L)	38.42 ± 3.37	33.37 ± 3.05	< 0.001
	BUN(mmol/L)	6.07 ± 1.41	7.54 ± 0.98	< 0.001
	Cr(µmol/L)	89.4 ± 17.7	105.0 ± 25.6	< 0.001
	GFR(ml/min)	104.58 ± 8.81	99.34 ± 12.46	0.023
	WBC(G/L)	4.63 ± 0.62	3.52 ± 0.73	< 0.001
	RBC(T/L)	4.53 ± 0.58	3.37 ± 0.74	< 0.001
	PLT(G/L)	124.67 ± 20.31	87.90 ± 23.32	< 0.001

TACE + sulitinib group: total bilirubin and WBC increased after treatment compared with those before treatment (P < 0.05); RBC and PLT decreased after treatment compared with those before treatment (P < 0.05); there was no statistically significant difference in Albumin before and after treatment (P = 0.091).

Sunitinib group: total bilirubin increased after treatment compared with that before treatment (P < 0.05); Albumin, WBC, RBC and PLT decreased after treatment compared with that before treatment (P < 0.05).

### Changes in renal function and ECOG before and after treatment in both groups

(1) Comparison of ECOG scores, renal function after treatment between the two groups(Table 2).

There were no statistical differences in ECOG scores, BUN, Cr and GFR after treatment between the two groups.(P > 0.05).

(2) Compare the changes of renal function before and after treatment in each group;(Table 3).

TACE + Sunitinib group: BUN and Cr after treatment increased compared with those before treatment (P < 0.05); GFR after treatment decreased compared with that before treatment (P < 0.05).

Sunitinib group: BUN and Cr increased after treatment compared with those before treatment (P < 0.05); GFR decreased after treatment compared with that before treatment (P < 0.05).

### Efficacy evaluation of tumors after treatment in the two groups (Table 4)

**Table 4:** Evaluation of tumor response after treatment in the two groups

			Group		Chi-Square Tests(p-value)	
			TACE + Sunitinib group(N = 47)	Sunitinib group(N = 51)	Pearson Chi-Square	Fisher’s Exact Test
Tumor response	CR	Count(%)	4(8.5%)	0(0%)	0.017	
	PR	Count(%)	27(57.5%)	20(39.2%)		
	SD	Count(%)	9(19.1%)	14(27.5%)		
	PD	Count(%)	7(14.9%)	17(33.3%)		
ORR		Count(%)	31(66.0%)	20(39.2%)		0.009
DCR		Count(%)	40(85.1%)	34(66.7%)		0.038

There was statistical difference in tumor response between the two groups after treatment(P < 0.05). Compared TACE + sunitinib group and sunitinib group, ORR was 66.0% vs. 39.2% (P = 0.009), DCR was 85.1% vs. 66.7% (P = 0.038), with statistical difference. (Table 4)

### PFS and OS of patients in the two groups(Table 5)

**Table 5:** Comparison of OS and PFS between the two groups

	Gruop	Median(months)	95% Confidence Interval		Log Rank (Mantel-Cox) (p-value)
			Lower Bound	Upper Bound	
PFS	TACE + Sunitinib group	15.6	14.9	17.1	< 0.001
	Sunitinib group	10.9	8.9	11.1	
OS	TACE + Sunitinib group	35.0	32.7	37.4	< 0.001
	Sunitinib group	25.7	23.6	27.8	

**Fig. 2:**
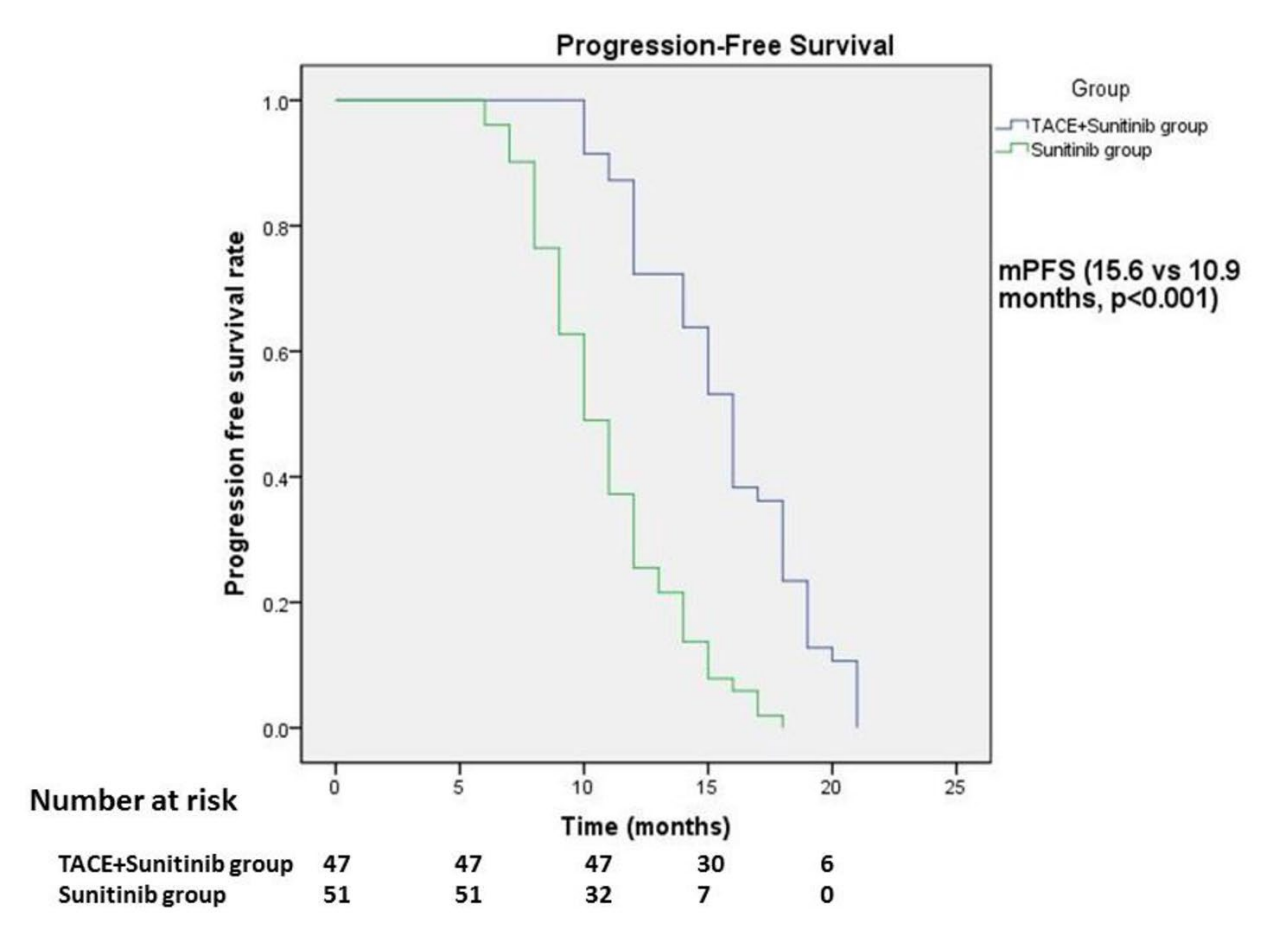
Progression-free survival time in the two groups mPFS: TACE + Sunitinib group, 15.6 months (95% CI 14.9–17.1 months); Sunitinib group, 10.9 months (95% CI 8.9–11.1 months).

**Fig. 3:**
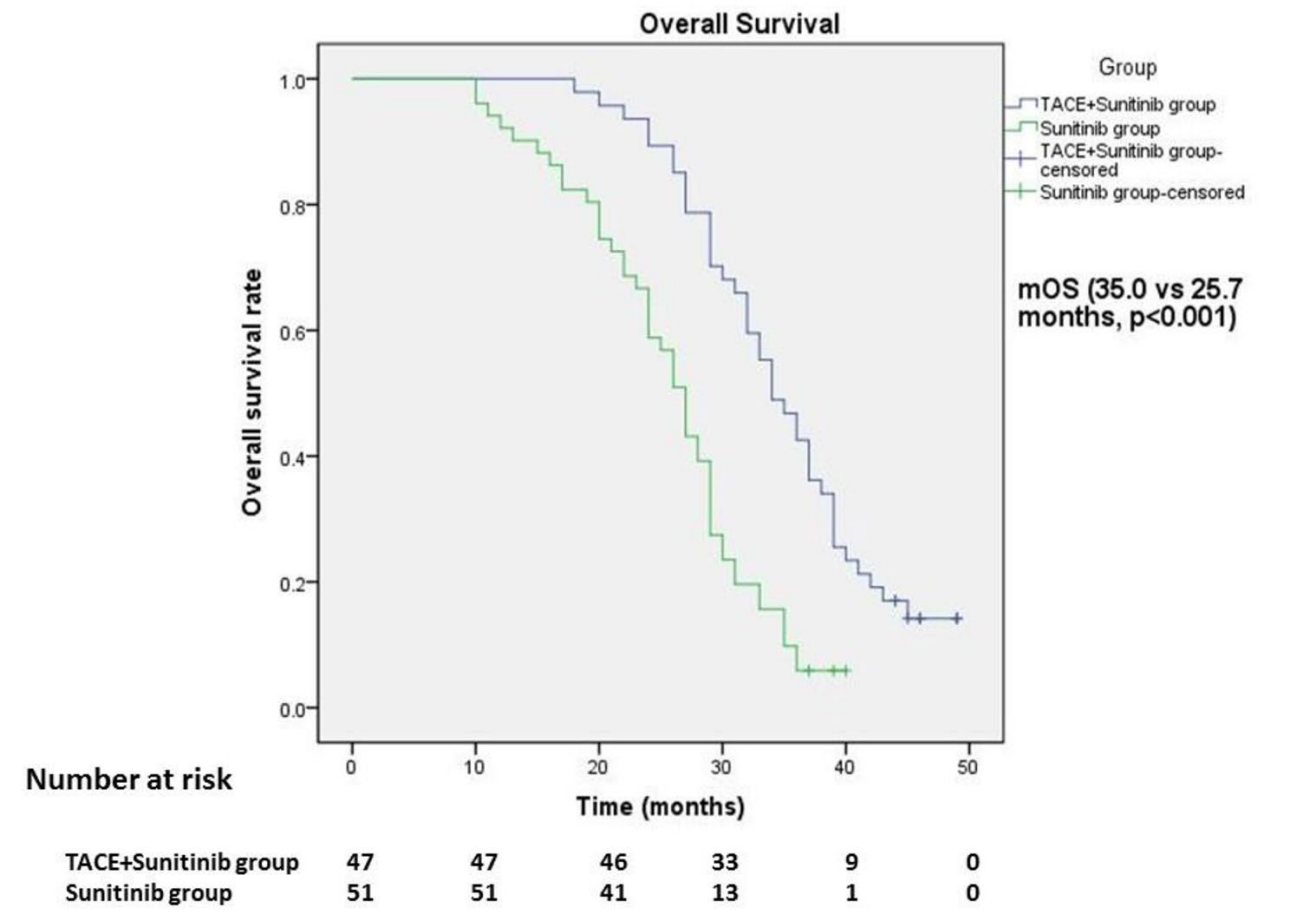
Overall survival of patients in two groups mOS: TACE + Sunitinib group, 35.0 months (95% CI 32.7–37.4 months); Sunitinib group, 25.7 months (95% CI 23.6–27.8 months).

Compared with TACE + sunitinib group and sunitinib group, mPFS was 15.6 months vs. 10.9 months (p < 0.001, Fig. 2; Table 5) and mOS was 35.0 months vs. 25.7 months (p < 0.001, Fig. 3; Table 5), with statistical difference.

### Incidence of adverse events after treatment in the two groups(Table 6)

**Table 6:** Incidence of treatment-related adverse reactions in the two groups

	TACE + Sunitinib group(N = 47)		Sunitinib group(N = 51)		Comparison of AEs(All)
	AEs	AEs(Grade 3–4)	AEs	AEs(Grade 3–4)	Chi-square test (p-value)
Abdominal pain	26(55.3%)	1(2.1%)	7(13.7%)	0	< 0.001
Fever	29(61.7%)	2(4.3%)	4(7.8%)	0	< 0.001
Vomiting	19(40.4%)	0	10(19.6%)	0	0.024
Asthenia	27(57.4%)	0	29(56.9%)	1(2.0%)	0.953
Decreased appetite	21(44.7%)	0	24(47.1%)	1(2.0%)	0.813
Hypertension	18(38.3%)	1(2.1%)	16(31.4%)	0	0.472
Hand-foot syndrome	20(42.6%)	0	21(41.2%)	1(2.0%)	0.890
Diarrhea	14(29.8%)	0	12(23.5%)	0	0.483
Rash	8(17.0%)	0	6(11.8%)	0	0.458

Adverse events were evaluated using Common Terminology Criteria for Adverse Events (CTCAE 5.0). The incidence of abdominal pain, fever, and vomiting was significantly higher in the TACE + sunitinib group than in the sunitinib group (abdominal pain: 55.3% vs. 13.7%; fever: 61.7% vs. 7.8%; vomiting: 40.4% vs. 19.6%; P < 0.05). There was no significant difference in the incidence of fatigue, anorexia, hypertension, hand-foot syndrome, diarrhea and rash between the TACE + sunitinib group and the sunitinib group (Table 6, P > 0.05). In TACE + Sunitinib group, 4(8.5%) patients’ doses were halved due to adverse events. In Sunitinib group, 3(5.9%) patients’ doses were halved due to adverse events.

## Discussion

About 70% of patients present with localized renal cell carcinoma(RCC) or locally advanced RCC, but about 30% of these patients experience recurrence or metastasis within 3 years after surgery [20]. At present, systemic drug therapy is the main treatment for Metastatic renal cell carcinoma (mRCC), including targeted drug therapy and immunotherapy [21].Before the advent of molecular targeted drugs, biological immunotherapy with interleukin-2 (IL-2) and interferon-α (IFN-α) was mainly used for advanced RCC [22], with an objective response rate of less than 30%, an mPFS of only 5 months [23], and a five-year survival rate of 8% for patients with mRCC [24]. With the application of a variety of targeted drugs in the treatment of RCC, the prognosis of patients with advanced RCC has improved significantly. According to the mechanism of action, targeted drugs are mainly divided into three types: upstream inhibitors targeting the mTOR pathway in tumors [25], such as Everolimus, Sirolimus, etc.; intermediate monoclonal antibodies targeting VEGF-A secreted by tumor cells, such as Bevacizumab, etc. [26]; downstream tyrosine kinase inhibitors targeting VEGFR/PDGFR and other receptors on vascular endothelial cells [27], such as Sorafenib, Sunitinib, Pazopanib, etc. Sunitinib is a multi-target receptor tyrosine kinase inhibitor [28], with the main targets of vascular endothelial growth factor receptor 1–2 (VEGFR1-2), platelet-derived growth factor receptor (PDGFR-α, PDGFR-β), stem cell growth factor receptor (c-KIT) and FMS-like tyrosine kinase 3 (FLT-3). It has the effects of anti-tumor angiogenesis and inhibiting tumor cell proliferation, thereby inhibiting the occurrence and metastasis of tumors. It is one of the most commonly used first-line drugs for the treatment of RCC at present. Michael Moran et al. reported [29] that in both randomized controlled trials(RCTs) and Real-World Data(RWD), sunitinib was an effective first-line treatment strategy for mRCC, with a mPFS of 7.5–11.0 months in RWD and 5.6–15.1 months in RCTs reported in the literature. The ORR was 14.0-34.6% in RWD and 18.8–46.9% in RCTs. The mOS was 6.8–33.2 months in RWD and 21.8–31.5 months in RCTs. Xiu-Lan Liu et al. reported in the study [30] that the treatment of RCC with sunitinib had better efficacy and safety than sorafenib. Our findings were an ORR of 39.2% and a DCR of 66.7% in the sunitinib group. In the sunitinib group, mPFS was 10.9 months (95% CI 8.9–11.1) and mOS was 25.7 months (95% CI 23.6–27.8). This result is consistent with other studies that treatment with sunitinib improves survival in patients with advanced RCC.

TACE is currently one of the commonly used means for the treatment of various solid tumors throughout the body. TACE has been used in hepatocellular carcinoma for about forty years. With the improvement of interventional techniques and the development of interventional devices (including embolic agents), TACE is also more and more widely used in the treatment of RCC [31]. For patients with pain, hematuria or tumor rupture and hemorrhage, TACE can effectively relieve patients’ clinical symptoms and play a role in hemostasis. Bryan Wright et al reported [32] that transarterial embolization (TAE) can effectively improve the symptoms of patients with RCC, such as pain and hematuria, and is a safe treatment strategy. 60 patients treated for pain and hematuria were reported in the study, with improvement in pain in 59 patients (98.3%) and improvement in hematuria in 57 patients (95%) after TAE. For advanced RCC that cannot be surgically removed, TACE can effectively and rapidly reduce the tumor burden [33]. The injection of chemotherapeutic drugs and embolic agents into the tumor site through the catheter has a variety of advantages [34]: (1) the total use of chemotherapeutic drugs is less than that of systemic chemotherapy, and the incidence of chemotherapy-related toxicities is low; (2) the concentration of chemotherapeutic drugs in the tumor site is high, which can better eliminate tumor cells; (3) the combined use of embolic agents by chemotherapeutic drug-lipiodol emulsion can slow down the loss of chemotherapeutic drugs, the local chemotherapeutic drugs are slowly released, and the effective drug concentration can be maintained in the tumor site for a long time; (4) after embolization of the tumor feeding artery, the ischemic necrosis of tumor tissue is more obvious, which can effectively reduce the tumor burden in a short time. For renal artery chemoembolization, there are also corresponding operating specifications [35]. Referring to the chemotherapy regimen for renal cancer, doxorubicin is one of the commonly used drugs [36] and the most classically used chemotherapeutic drug for TACE for liver cancer. The Nathaniel R’ study [37] suggested that the combination of doxorubicin with epigenetic therapeutics can be beneficial in clinical treatment of renal cancer patients with wild-type VHL and p53. Therefore, the chemotherapeutic drug used in chemoembolization of RCC in our center is doxorubicin. J H Park et al reported[38] that for patients with inoperable RCC, TAE using lipiodol + ethanol emulsion was an effective and safe treatment. Noor Riza Perdana et al reported [39] that RAE is an effective treatment for large unresectable renal tumors and can reduce mortality. T Kato et al reported [40] that chemoembolization with mitomycin C microcapsules is a very effective treatment for renal cell carcinoma. It is reported by H Saitoh et al [41] that the use of renal artery embolization in the treatment of advanced RCC is an effective translational treatment, which can reduce the tumor burden, increase the chance of surgery for patients, and improve the survival of patients. A M Granov et al reported [42] that the use of chemoembolization for advanced RCC has a higher 2-year and 3-year survival rate relative to embolization alone. The results of this study showed that in TACE + Sunitinib group, CR in 4 patients (8.5%), PR in 27 patients (57.5%), SD in 9 patients (19.1%) and PD in 7 patients (14.9%); ORR was 66.0% and DCR was 85.1%. TACE is similar to surgical cytoreductive surgery in reducing the tumor burden of RCC, but TACE is more minimally invasive and patients experience less pain and trauma.

Numerous studies have reported [43] that tumor tissue hypoxia leads to a significant increase in VEGF levels in patients with hepatocellular carcinoma(HCC) after receiving TACE therapy. Similar to HCC, tumor tissue ischemia and hypoxia in patients with RCC induce increased hypoxia-inducible factor(HIF) activity after TACE, which in turn leads to overexpression of various tumor-promoting factors such as vascular endothelial growth factor (VEGF), transforming growth factor (TGF), and platelet-derived growth factor (PDGF) [18]. The overexpression of these cytokines leads to cell proliferation, apoptosis inhibition, angiogenesis, and increased adhesion and mobility, which in turn leads to tumor development [1, 44, 45]. Molecular targeted drugs can target and inhibit these cytokine receptors, which can compensate for the lack after TACE treatment in a mechanistic manner, thereby improving the effect of TACE treatment. TACE combined with molecular targeted drug therapy has been very explored in the treatment of HCC, and there are also a large number of literatures reporting that combination therapy can effectively improve the deficiencies of TACE [46]. Masatoshi Kudo study reported [47] that TACE plus sorafenib significantly improved PFS over TACE alone in patients with unresectable HCC. It is reported by Zhigang Fu et al[48] that Combination treatment with TACE and lenvatinib may improve clinical outcomes over TACE monotherapy with a manageable safety profile for unresectable HCC. Although TACE for RCC has not been reported in combination therapy. However, based on the experience of TACE combined with molecular targeted drugs in the treatment of HCC, our center also uses molecular targeted drug Sunitinib in patients with advanced RCC after TACE. This study found that in the TACE + sunitinib group, mPFS was 15.6 months (95% CI 14.9–17.1); mOS was 35.0 months (95% CI 32.7–37.4). PFS and OS were significantly longer in the TACE + sunitinib group than in the sunitinib-only group, and patients had a better survival benefit.

The current guidelines recommend a dose of 50 mg qd for sunitinib, 4/2 regimen, that is 4 weeks of treatment followed by 2 weeks of rest as a cycle. Some scholars have also proposed a 2/1 regimen after research, that is treatment with 50 mg qd for 2 weeks, followed by 1 week of rest. Some patients may experience AEs, such as hand-foot syndrome, fatigue, leukopenia, hypertension, thrombocytopenia, and anemia. S Bracarda et al. reported [49] that patients treated with 2/1 regimen had similar efficacy, but the incidence of side effects was significantly reduced. In this study, 50 mg qd(4/2 regimen) was used for sulitinib. There was no statistical difference in the incidence rate of fatigue, anorexia, hypertension, hand-foot syndrome, diarrhea and rash between the two groups (P > 0.05), and the incidence rate of grade 3–4 AEs was very low in the two groups. However, the incidence of abdominal pain, fever, and vomiting was significantly higher in the TACE + sunitinib group than in the sunitinib group (abdominal pain: 55.3% vs. 13.7%; fever: 61.7% vs. 7.8%; vomiting: 40.4% vs. 19.6%; P < 0.05). The main reason was the occurrence of post-embolization syndrome after TACE in the combined treatment group. T Onishi et al. reported [50] that the most important AEs after TAE for unresectable advanced RCC were abdominal pain, fever, nausea and vomiting, and all patients in the study recovered from the AEs. Most post-embolization syndromes can be relieved in a short time after symptomatic treatment. Yonghua Bi et al. [51] reported that 35 patients with unresectable RCC treated with TACE developed fever, abdominal pain, nausea and vomiting, and these symptoms were relieved after 2–3 days. It is reported by Shu-Kui Qin et al. [52] that in the first-line treatment of mRCC with sunitinib, the most common AEs in the Chinese population were hand-foot syndrome (63.8%), leukopenia (52.4%), fatigue (51.4%) and thrombocytopenia (51.4%), all of which were tolerable, and AEs predicted longer PFS and OS. There was no statistical difference in ECOG score after treatment between the two groups in this study (P > 0.05). After treatment, total bilirubin, BUN and Cr in the two groups were significantly higher than those before treatment. After treatment, GFR, WBC and PLT in the two groups were lower than those before treatment, but there was no statistical difference between the two groups (P > 0.05). In TACE + Sunitinib group, WBC increased after treatment compared with that before treatment, which was considered to be ischemic necrosis of tumor tissue after TACE, and aseptic inflammation caused by necrotic tissue, thus WBC increased compared with that before treatment. While in Sunitinib group, WBC decreased after treatment compared with that before treatment, which might be considered to be AEs caused by the drug. Albumin decreased after TACE + sunitinib group and sunitinib group compared with that before treatment, and decreased more in sunitinib group, with statistical difference (P < 0.05). After analysis, 14 patients (27.5%) had SD and 17 (33.3%) had PD in Sunitinib group. Their disease control rate was worse than TACE + Sunitinib group, which might be associated with decreased liver function due to disease progression. The results of this study showed that the combination therapy did not increase the risk of other treatment-related AEs except that the incidence of post-embolization syndrome which was higher in the TACE + sunitinib group than in the sunitinib group.

The shortcomings of this study are that the data are from a single center, and it is a retrospective study with limited sample size. A multicenter, large-sample, prospective study is feasible at a later stage to provide more help for clinical work.

## Conclusions

For advanced renal cell carcinoma that cannot be surgically removed, TACE is able to effectively reduce the tumor burden of patients. The TACE + sunitinib group had a higher ORR (66.0% vs. 39.2%) and DCR (85.1% vs. 66.7%) than the sunitinib alone group. TACE combined with sunitinib in the treatment of unresectable advanced RCC can obtain longer PFS (mPFs: 15.6 months) and OS (mOS: 35.0 months). TACE + sunitinib is as safe as sunitinib and does not increase the incidence of sunitinib-related AEs. Therefore, TACE combined with sunitinib can play a complementary role and is a safe and effective treatment for advanced RCC.

## Data Availability

The datasets used and analysed during the current study are available from the corresponding author on reasonable request.

## References

[1] Randall JM, Millard F, Kurzrock R. Molecular aberrations, targeted therapy, and renal cell carcinoma: current state-of-the-art. Cancer Metastasis Rev. 2014 Dec;33(4):1109-24. doi: 10.1007/s10555-014-9533-1. PMID: 25365943. 10.1007/s10555-014-9533-1 25365943

[2] Spadaccino F, Netti GS, Rocchetti MT, Castellano G, Stallone G, Ranieri E. [Diagnostic and prognostic markers of renal cell carcinoma]. G Ital Nefrol. 2020 Apr 9;37(2):2020-vol2. Italian. PMID: 32281759. 32281759

[3] Pullen RL Jr. Renal cell carcinoma, part 1. Nursing. 2021 Jul 1;51(7):34–40. doi: 10.1097/01.NURSE.0000753972.19135.dc. PMID: 34156999. 10.1097/01.NURSE.0000753972.19135.dc 34156999

[4] Huang J, Leung DK, Chan EO, Lok V, Leung S, Wong I, Lao XQ, Zheng ZJ, Chiu PK, Ng CF, Wong JH, Volpe A, Merseburger AS, Powles T, Teoh JY, Wong MCS. A Global Trend analysis of kidney Cancer incidence and mortality and their Associations with Smoking, Alcohol Consumption, and metabolic syndrome. Eur Urol Focus. 2022 Jan;8(1):200–9. Epub 2021 Jan 23. PMID: 33495133. 10.1016/j.euf.2020.12.020 33495133

[5] Linehan WM, Ricketts CJ. The Cancer Genome Atlas of renal cell carcinoma: findings and clinical implications. Nat Rev Urol. 2019 Sep;16(9):539–552. doi: 10.1038/s41585-019-0211-5. Epub 2019 Jul 5. PMID: 31278395. 10.1038/s41585-019-0211-5 31278395

[6] Mendhiratta N, Muraki P, Sisk AE Jr, Shuch B. Papillary renal cell carcinoma: review. Urol Oncol. 2021 Jun;39(6):327–37. 10.1016/j.urolonc.2021.04.013. Epub 2021 May 24. PMID: 34034966. 10.1016/j.urolonc.2021.04.013 34034966

[7] Garje R, Elhag D, Yasin HA, Acharya L, Vaena D, Dahmoush L. Comprehensive review of chromophobe renal cell carcinoma. Crit Rev Oncol Hematol. 2021 Apr;160:103287. 10.1016/j.critrevonc.2021.103287. Epub 2021 Mar 19. PMID: 33753250. 10.1016/j.critrevonc.2021.103287 33753250

[8] Ljungberg B, Albiges L, Abu-Ghanem Y, Bensalah K, Dabestani S, Fernández-Pello S, Giles RH, Hofmann F, Hora M, Kuczyk MA, Kuusk T, Lam TB, Marconi L, Merseburger AS, Powles T, Staehler M, Tahbaz R, Volpe A, Bex A. European Association of Urology Guidelines on Renal Cell Carcinoma: the 2019 Update. Eur Urol. 2019 May;75(5):799–810. Epub 2019 Feb 23. PMID: 30803729. 10.1016/j.eururo.2019.02.011 30803729

[9] Escudier B, Porta C, Schmidinger M, Rioux-Leclercq N, Bex A, Khoo V, Grünwald V, Gillessen S, Horwich A, ESMO Guidelines Committee. ;. Electronic address: clinicalguidelines@esmo.org. Renal cell carcinoma: ESMO Clinical Practice Guidelines for diagnosis, treatment and follow-up†. Ann Oncol. 2019 May 1;30(5):706–720. doi: 10.1093/annonc/mdz056. PMID: 30788497. 10.1093/annonc/mdz056 30788497

[10] Liu Z, Li Y, Zhao X, Ge L, Zhu G, Hong P, Tang S, Zhang S, Tian X, Wang S, Liu C, Zhang H, Ma L. Renal cell carcinoma with tumor thrombus growing against the direction of venous return: an indicator of complicated surgery and poor prognosis. BMC Surg. 2021 Dec 28;21(1):443. doi: 10.1186/s12893-021-01448-0. PMID: 34963464; PMCID: PMC8713414. 10.1186/s12893-021-01448-0 PMC8713414 34963464

[11] Chen Z, Yang F, Ge L, Qiu M, Liu Z, Liu C, Tian X, Zhang S, Ma L. Outcomes of renal cell carcinoma with associated venous tumor thrombus: experience from a large cohort and short time span in a single center. BMC Cancer. 2021 Jul 2;21(1):766. doi: 10.1186/s12885-021-08508-x. PMID: 34215223; PMCID: PMC8254310. 10.1186/s12885-021-08508-x PMC8254310 34215223

[12] Escudier B, Porta C, Schmidinger M, Rioux-Leclercq N, Bex A, Khoo V, Gruenvald V, Horwich A, ESMO Guidelines Committee. ;. Renal cell carcinoma: ESMO Clinical Practice Guidelines for diagnosis, treatment and follow-up. Ann Oncol. 2016 Sep;27(suppl 5):v58-v68. doi: 10.1093/annonc/mdw328. Erratum in: Ann Oncol. 2017 Jul 1;28(suppl_4):iv167-iv168. PMID: 27664262. 10.1093/annonc/mdw328 27664262

[13] Quhal F, Mori K, Bruchbacher A, Resch I, Mostafaei H, Pradere B, Schuettfort VM, Laukhtina E, Egawa S, Fajkovic H, Remzi M, Shariat SF, Schmidinger M. First-line Immunotherapy-based Combinations for Metastatic Renal Cell Carcinoma: A Systematic Review and Network Meta-analysis.Eur Urol Oncol. 2021Oct;4(5):755–765. doi: 10.1016/j.euo.2021.03.001. Epub 2021 Mar 20. PMID: 33757737. 10.1016/j.euo.2021.03.001 33757737

[14] Motzer RJ, Escudier B, McDermott DF, Arén Frontera O, Melichar B, Powles T, Donskov F, Plimack ER, Barthélémy P, Hammers HJ, George S, Grünwald V, Porta C, Neiman V, Ravaud A, Choueiri TK, Rini BI, Salman P, Kollmannsberger CK, Tykodi SS, Grimm MO, Gurney H, Leibowitz-Amit R, Geertsen PF, Amin A, Tomita Y, McHenry MB, Saggi SS, Tannir NM. Survival outcomes and independent response assessment with nivolumab plus ipilimumab versus sunitinib in patients with advanced renal cell carcinoma: 42-month follow-up of a randomized phase 3 clinical trial. J Immunother Cancer. 2020 Jul;8(2):e000891. doi: 10.1136/jitc-2020-000891. Erratum in: J Immunother Cancer. 2021 May;9(5): PMID: 32661118; PMCID: PMC7359377. 10.1136/jitc-2020-000891 PMC7359377 32661118

[15] Singla N, Hutchinson RC, Ghandour RA, Freifeld Y, Fang D, Sagalowsky AI, Lotan Y, Bagrodia A, Margulis V, Hammers HJ, Woldu SL. Improved survival after cytoreductive nephrectomy for metastatic renal cell carcinoma in the contemporary immunotherapy era: an analysis of the National Cancer Database. Urol Oncol. 2020 Jun;38(6):604. .e9-604.e17. Epub 2020 Apr 3. PMID: 32253116; PMCID: PMC7269798. 10.1016/j.urolonc.2020.02.029 PMC7269798 32253116

[16] Yamada R, Bassaco B, Bracewell S, Gillen K, Kocher M, Collins H, Anderson MB, Guimaraes M. Long-term follow-up after conventional transarterial chemoembolization (c-TACE) with mitomycin for hepatocellular carcinoma (HCC). J Gastrointest Oncol. 2019 Apr;10(2):348–53. 10.21037/jgo.2019.01.01. PMID: 31032104; PMCID: PMC6465494. 10.21037/jgo.2019.01.01 PMC6465494 31032104

[17] Gunn AJ, Patel AR, Rais-Bahrami S. Role of Angio-Embolization for Renal Cell Carcinoma. Curr Urol Rep. 2018 Aug 8;19(10):76. doi: 10.1007/s11934-018-0827-7. PMID: 30091047. 10.1007/s11934-018-0827-7 30091047

[18] Sakr OS, Berndt S, Carpentier G, Cuendet M, Jordan O, Borchard G. Arming embolic beads with anti-VEGF antibodies and controlling their release using LbL technology. J Control Release. 2016 Feb 28;224:199–207. doi: 10.1016/j.jconrel.2016.01.010. Epub 2016 Jan 11. PMID: 26780173. 10.1016/j.jconrel.2016.01.010 26780173

[19] Zhou M, Zhang C, Nie J, Sun Y, Xu Y, Wu F, Huang Y, Li S, Wang Y, Zhou Y, Zheng T. Response Evaluation and Survival Prediction Following PD-1 Inhibitor in Patients With Advanced Hepatocellular Carcinoma: Comparison of the RECIST 1.1, iRECIST, and mRECIST Criteria.Front Oncol. 2021 Dec9;11:764189. doi: 10.3389/fonc.2021.764189. PMID: 34956885; PMCID: PMC8697350. 10.3389/fonc.2021.764189 PMC8697350 34956885

[20] Wiechno P, Kucharz J, Sadowska M, Michalski W, Sikora-Kupis B, Jonska-Gmyrek J, Poniatowska G, Nietupski K, Ossolinski K, Demkow T. Contemporary treatment of metastatic renal cell carcinoma. Med Oncol. 2018 Oct 27;35(12):156. doi: 10.1007/s12032-018-1217-1. PMID: 30368624. 10.1007/s12032-018-1217-1 30368624

[21] Hahn AW, Klaassen Z, Agarwal N, Haaland B, Esther J, Ye XY, Wang X, Pal SK, Wallis CJD. First-line Treatment of Metastatic Renal Cell Carcinoma: A Systematic Review and Network Meta-analysis. Eur Urol Oncol. 2019 Nov;2(6):708–715. doi: 10.1016/j.euo.2019.09.002. Epub 2019 Oct 4. PMID: 31588018. 10.1016/j.euo.2019.09.002 31588018

[22] McDermott DF, Atkins MB. Application of IL-2 and other cytokines in renal cancer. Expert Opin Biol Ther. 2004 Apr;4(4):455 – 68. doi: 10.1517/14712598.4.4.455. PMID: 15102596. 10.1517/14712598.4.4.455 15102596

[23] de Velasco G, Hamieh L, Mickey S, Choueiri TK. Optimizing systemic therapy for metastatic renal cell carcinoma beyond the first-line setting. Urol Oncol. 2015 Dec;33(12):538–45. 10.1016/j.urolonc.2015.08.007. Epub 2015 Oct 9. PMID: 26482392; PMCID: PMC4654640. 10.1016/j.urolonc.2015.08.007 PMC4654640 26482392

[24] Choueiri TK, Motzer RJ. Systemic Therapy for Metastatic Renal-Cell Carcinoma. N Engl J Med. 2017 Jan 26;376(4):354–366. doi: 10.1056/NEJMra1601333. PMID: 28121507. 10.1056/NEJMra1601333 28121507

[25] Gluskin J, Plodkowski A, Girshman J, Sarasohn D, Viteri-Jusué A, Hayan S, Torrisi J. Waxing and waning pattern of mTOR inhibitor-associated pneumonitis in renal cell carcinoma patients: a retrospective observational study. Clin Imaging. 2021 Mar;71:29–33. 10.1016/j.clinimag.2020.10.052. Epub 2020 Nov 5. PMID: 33171363; PMCID: PMC7855089. 10.1016/j.clinimag.2020.10.052 PMC7855089 33171363

[26] McDermott DF, George DJ. Bevacizumab as a treatment option in advanced renal cell carcinoma: an analysis and interpretation of clinical trial data. Cancer Treat Rev. 2010 May;36(3):216–23. 10.1016/j.ctrv.2009.12.003. Epub 2010 Jan 29. PMID: 20116176. 10.1016/j.ctrv.2009.12.003 20116176

[27] Roskoski R Jr. Vascular endothelial growth factor (VEGF) and VEGF receptor inhibitors in the treatment of renal cell carcinomas.Pharmacol Res. 2017 Jun;120:116–132. doi: 10.1016/j.phrs.2017.03.010. Epub 2017 Mar 19. PMID: 28330784. 10.1016/j.phrs.2017.03.010 28330784

[28] Badran A, Elshenawy MA, Shahin A, Aljubran A, Alzahrani A, Eldali A, Bazarbashi S. Efficacy and Prognostic Factors of Sunitinib as First-Line Therapy for Patients With Metastatic Renal Cell Carcinoma in an Arab Population.JCO Glob Oncol. 2020 Feb;6:19–26. doi: 10.1200/JGO.19.00111. PMID: 32031432; PMCID: PMC6998020. 10.1200/JGO.19.00111 PMC6998020 32031432

[29] Moran M, Nickens D, Adcock K, Bennetts M, Desscan A, Charnley N, Fife K. Target Oncol. 2019 Aug;14(4):405–16. PMID: 31301015; PMCID: PMC6684538. Sunitinib for Metastatic Renal Cell Carcinoma: A Systematic Review and Meta-Analysis of Real-World and Clinical Trials Data. 10.1007/s11523-019-00653-5 PMC6684538 31301015

[30] Liu XL, Xue HY, Chu Q, Liu JY, Li J. Comparative efficacy and safety of sunitinib vs sorafenib in renal cell carcinoma: A systematic review and meta-analysis. Medicine (Baltimore). 2020 Mar;99(13):e19570. doi: 10.1097/MD.0000000000019570. PMID: 32221075; PMCID: PMC7220148. 10.1097/MD.0000000000019570 PMC7220148 32221075

[31] Ramaswamy RS, Darcy MD. Arterial Embolization for the Treatment of Renal Masses and Traumatic Renal Injuries. Tech Vasc Interv Radiol. 2016 Sep;19(3):203 – 10. doi: 10.1053/j.tvir.2016.06.005. Epub 2016 Jun 3. PMID: 27641454. 10.1053/j.tvir.2016.06.005 27641454

[32] Wright B, Johnson BS, Vassar M, Saidian A, Rais-Bahrami S, Gunn AJ. Trans-arterial embolization of renal cell carcinoma: a systematic review and meta-analysis. Abdom Radiol (NY). 2022 Apr 5. doi: 10.1007/s00261-022-03502-8. Epub ahead of print. PMID: 35380246. 10.1007/s00261-022-03502-8 35380246

[33] Duan XH, Li YS, Han XW, Wang YL, Jiao DC, Li TF, Chen PF, Fang Y. C-arm CT-guided renal arterial embolisation followed by radiofrequency ablation for treatment of patients with unresectable renal cell carcinoma. Clin Radiol. 2016 Jan;71(1):79–85. Epub 2015 Nov 18. PMID: 26602936. 10.1016/j.crad.2015.10.012 26602936

[34] Wallace S, Charnsangavej C, Carrasco CH, Bechtel W, Infusion-embolization. Cancer. 1984 Dec 1;54(11 Suppl):2751-65. doi: 10.1002/1097-0142(19841201)54:2+>2751::aid-cncr2820541423<3.0.co;2-5. PMID: 6093984. 10.1002/1097-0142(19841201)54:2+<2751::aid-cncr2820541423>3.0.co;2-5 6093984

[35] Ramaswamy RS, Akinwande O, Tiwari T. Renal Embolization: Current Recommendations and Rationale for Clinical Practice. Curr Urol Rep. 2018 Feb 5;19(3):5. doi: 10.1007/s11934-018-0756-5. PMID: 29399726. 10.1007/s11934-018-0756-5 29399726

[36] Haas NB, Lin X, Manola J, Pins M, Liu G, McDermott D, Nanus D, Heath E, Wilding G, Dutcher J. A phase II trial of doxorubicin and gemcitabine in renal cell carcinoma with sarcomatoid features: ECOG 8802. Med Oncol. 2012 Jun;29(2):761–7. 10.1007/s12032-011-9829-8. Epub 2011 Feb 6. PMID: 21298497; PMCID: PMC3566570. 10.1007/s12032-011-9829-8 PMC3566570 21298497

[37] Acharya N, Singh KP. Differential sensitivity of renal carcinoma cells to doxorubicin and epigenetic therapeutics depends on the genetic background. Mol Cell Biochem. 2021 Jun;476(6):2365–79. 10.1007/s11010-021-04076-7. Epub 2021 Feb 16. PMID: 33591455. 10.1007/s11010-021-04076-7 33591455

[38] Park JH, Kim SH, Han JK, Chung JW, Han MC. Transcatheter arterial embolization of unresectable renal cell carcinoma with a mixture of ethanol and iodized oil. Cardiovasc Intervent Radiol. 1994 Nov-Dec;17(6):323-7. doi: 10.1007/BF00203951. PMID: 7533666. 10.1007/BF00203951 7533666

[39] Perdana NR, Daulay ER, Prapiska FF. Renal Arteries Embolization in Unresectable Clear Cell Renal Carcinoma: First Time Experience at Haji Adam Malik Hospital. Open Access Maced J Med Sci. 2018 Aug 19;6(8):1454–1457. doi: 10.3889/oamjms.2018.282. PMID: 30159076; PMCID: PMC6108812. 10.3889/oamjms.2018.282 PMC6108812 30159076

[40] Kato T, Nemoto R, Mori H, Takahashi M, Tamakawa Y. Transcatheter arterial chemoembolization of renal cell carcinoma with microencapsulated mitomycin C. J Urol. 1981 Jan;125(1):19–24. doi: 10.1016/s0022-5347(17)54880-6. PMID: 7463576. 10.1016/s0022-5347(17)54880-6 7463576

[41] Saitoh H, Hayakawa K, Nishimura K, Kubo S, Hida S. Long-term results of ethanol embolization of renal cell carcinoma. Radiat Med. 1997 Mar-Apr;15(2):99–102. PMID: 9192434. 9192434

[42] Granov AM, Gorelov AI, Gershanovich ML, Karelin MI, Vorob’ev AV, Filov VA, Stukov AN (1998). Resul’taty primeneniia éndovaskuliarnykh vmeshatel’stv (émbolizatsii i khimioèmbolizatsii) v lechenii operabel’nogo i rasprostranennogo raka pochki [Results of endovascular interventions (embolization and chemoembolization) in the treatment of operable and extensive kidney cancer]. Vopr Onkol.

[43] Liu K, Min XL, Peng J, Yang K, Yang L, Zhang XM. The changes of HIF-1α and VEGF expression after TACE in patients with Hepatocellular Carcinoma. J Clin Med Res. 2016 Apr;8(4):297–302. 10.14740/jocmr2496w. Epub 2016 Feb 27. PMID: 26985249; PMCID: PMC4780492. 10.14740/jocmr2496w PMC4780492 26985249

[44] Daniela S, Krause,Richard A, Van Etten (2005). Tyrosine kinases as targets for cancer therapy. N Engl J Med.

[45] Gollob JA, Wilhelm S, Carter C, Kelley SL. Role of Raf kinase in cancer: therapeutic potential of targeting the Raf/MEK/ERK signal transduction pathway. Semin Oncol. 2006 Aug;33(4):392–406. doi: 10.1053/j.seminoncol.2006.04.002. PMID: 16890795. 10.1053/j.seminoncol.2006.04.002 16890795

[46] Deng J, Wen F. Transarterial Chemoembolization Combined With Tyrosine Kinase Inhibitors for Intermediate-Stage Hepatocellular Carcinoma, What Else Can We Do? Front Oncol. 2022 Mar 29;12:824799. doi: 10.3389/fonc.2022.824799. PMID: 35425716; PMCID: PMC9001928. 10.3389/fonc.2022.824799 PMC9001928 35425716

[47] Kudo M, Ueshima K, Ikeda M, Torimura T, Tanabe N, Aikata H, Izumi N, Yamasaki T, Nojiri S, Hino K, Tsumura H, Kuzuya T, Isoda N, Yasui K, Aino H, Ido A, Kawabe N, Nakao K, Wada Y, Yokosuka O, Yoshimura K, Okusaka T, Furuse J, Kokudo N, Okita K, Johnson PJ, Arai Y. ; TACTICS study group. Randomised, multicentre prospective trial of transarterial chemoembolisation (TACE) plus sorafenib as compared with TACE alone in patients with hepatocellular carcinoma: TACTICS trial.Gut. 2020Aug;69(8):1492–1501. doi: 10.1136/gutjnl-2019-318934. Epub 2019 Dec 4. PMID: 31801872; PMCID: PMC7398460. 10.1136/gutjnl-2019-318934 PMC7398460 31801872

[48] Fu Z, Li X, Zhong J, Chen X, Cao K, Ding N, Liu L, Zhang X, Zhai J, Qu Z. Lenvatinib in combination with transarterial chemoembolization for treatment of unresectable hepatocellular carcinoma (uHCC): a retrospective controlled study.Hepatol Int. 2021Jun;15(3):663–675. doi: 10.1007/s12072-021-10184-9. Epub 2021 Apr 20. PMID: 33877527; PMCID: PMC8286947. 10.1007/s12072-021-10184-9 PMC8286947 33877527

[49] Bracarda S, Iacovelli R, Boni L, Rizzo M, Derosa L, Rossi M, Galli L, Procopio G, Sisani M, Longo F, Santoni M, Morelli F, Di Lorenzo G, Altavilla A, Porta C, Camerini A, Escudier B, Rainbow Group. ;. Sunitinib administered on 2/1 schedule in patients with metastatic renal cell carcinoma: the RAINBOW analysis. Ann Oncol. 2016 Feb;27(2):366. doi: 10.1093/annonc/mdv589. Epub 2015 Dec 18. Erratum for: Ann Oncol. 2015 Oct;26(10):2107-13. PMID: 26685011. 10.1093/annonc/mdv315 26216384

[50] Onishi T, Oishi Y, Suzuki Y, Asano K. Prognostic evaluation of transcatheter arterial embolization for unresectable renal cell carcinoma with distant metastasis. BJU Int. 2001 Mar;87(4):312-5. doi: 10.1046/j.1464-410x.2001.00070.x. PMID: 11251521. 10.1046/j.1464-410x.2001.00070.x 11251521

[51] Bi Y, Shi X, Ren J, Yi M, Han X. Transarterial chemoembolization of unresectable renal cell carcinoma with doxorubicin-loaded CalliSpheres drug-eluting beads. Sci Rep. 2022 May 17;12(1):8136. doi: 10.1038/s41598-022-12334-x. PMID: 35581365; PMCID: PMC9113996. 10.1038/s41598-022-12334-x PMC9113996 35581365

[52] Qin SK, Jin J, Guo J, Wang JW, Zhou FJ, Huang YR, Ren XB, Ye DW, Pan S, Sajben P, Wang Q. Efficacy and safety of first-line sunitinib in chinese patients with metastatic renal cell carcinoma. Future Oncol. 2018 Aug;14(18):1835–45. 10.2217/fon-2017-0733. Epub 2018 May 2. PMID: 29717651. 10.2217/fon-2017-0733 29717651

